# Association of sex with the global burden of vision impairment caused by neonatal preterm birth: An analysis from the global burden of disease study 2019

**DOI:** 10.3389/fpubh.2022.938228

**Published:** 2022-07-27

**Authors:** Xin Ye, Jun Wang, Xiaxing Zhong, Wangli Qiu, Shangchao Yang, Shucheng He, Lixia Lou, Lijun Shen

**Affiliations:** ^1^School of Ophthalmology and Eye Hospital, Wenzhou Medical University, Wenzhou, China; ^2^Eye Center, School of Medicine, The Second Affiliated Hospital of Zhejiang University, Hangzhou, China

**Keywords:** neonatal preterm birth (NPB), Global Burden of Disease, years lived with disability (YLDs), Human Development Index (HDI), sex difference

## Abstract

**Aims:**

To investigate the sex-specific global burden of neonatal preterm birth (NPB) vision impairment by year, age, and socioeconomic status using years lived with disability (YLDs).

**Methods:**

The global, regional, and national sex-specific YLD numbers, crude YLD rates, and age-standardized YLD rates of NPB-related moderate and severe vision loss and blindness were obtained from the Global Burden of Disease Study 2019. The Wilcoxon test and linear regression were used to investigate the relationship between sex difference in age-standardized YLD rates and the Human Development Index (HDI).

**Results:**

Between 1990 and 2019, the gender disparity in age-standardized YLD rates for NPB-related vision impairment remained stable, increasing from 10.2 [95% uncertainty interval (UI) 6.7–14.6] to 10.4 (95% UI 6.9–15.0) for men and 10.3 (95% UI 6.8–14.7) to 10.7 (95% UI 7.2–15.1) for women, with women consistently having higher age-standardized YLD rates. Between the ages of 25 and 75, women had higher YLD rates than males, with the biggest disparity in the 60–64 age group. In 2019, sex difference in age-standardized YLD rates across 195 nations was statistically significant. Women had higher age-standardized YLD rates than men in both low **(***Z* = −3.53, *p* < 0.001**)** and very high HDI countries (*Z* = −4.75, *p* < 0.001). Additionally, age-standardized YLD rates were found to be adversely associated with HDI (male: Standardized β = −0.435, female: Standardized β = −0.440; *p* < 0.001).

**Conclusion:**

Despite advancements in worldwide NPB health care, sexual differences in NPB-related vision impairment burden showed little change. Female had higher burden than male, particularly in low and very high socioeconomic status countries.

## Key messages

- Despite advancements in worldwide NPB health care, sexual differences in NPB-related vision impairment burden showed little change.
- Female had higher burden than male, particularly in low and very high socioeconomic status countries.
- Our findings may enhance public awareness of sexual differences in global NPB-related vision impairment burden and reveal the importance of developing sex-sensitive health policy to manage global vision loss caused by NPB.

## Introduction

Neonatal preterm birth (NPB) is defined as livebirth before 37 completed weeks of gestation (1). NPB is a common neonatal disorder affecting ~15 million infants worldwide (2). A study identified 1,241 data points across 107 countries and estimated global preterm birth rate for 2014 to be 10.6%, equating to an estimated 14.84 million live preterm births in 2014. Asia and sub-Saharan Africa account for 12 million of these preterm births (1). The Global Burden of Disease (GBD) study used years lived with disability (YLDs) to assess the health burden of NPB. In 2019, NPB caused 664,000 deaths and contributed to 13.2% of all deaths in children under five (3). Additionally, NPB was responsible for 3.3% of YLDs among children and adolescents (<20 years old), placing it as the sixth-ranked Level 4 cause of disability. NPB-related vision impairment is of great significance to the quality of life, social economy, and public health of countries and regions (4). In 2019, NPB caused 822 million YLDs in vision impairment (including blindness, severe vision loss, and moderate vision loss) and led to 3.1% of all cause-related vision impairment YLDs of all ages. Females are more likely to suffer from vision impairment than males, with the YLDs being 453 million in females and 369 million in males, according to the GBD study 2019. Several biological, affective, cognitive, and sociocultural factors have been suggested to contribute to female vulnerability to vision impairment.

Blindness and vision loss (BVL) are the accumulation of different stages of vision loss. NPB-related blindness may be due to retinopathy of prematurity (ROP), congenital cataract, and congenital glaucoma. ROP is a proliferative vitreoretinopathy that occurs in premature infants and is the leading cause of childhood blindness worldwide (5). Premature infants also tend to have a higher risk of congenital cataracts, and the incidence is 0.97–1.9% (6). Primary congenital glaucoma is also more common in premature infants. According to a 2006 study, congenital glaucoma incidence is much higher in the preterm neonate population (2%) than in the general population (7). Premature infants also have been linked to an increased prevalence of strabismus, myopia, lower stereoacuity, and peripheral vision loss. It is reported that significant refractive errors were found in 29.6% of the preterm birth and 7.8% of term-born children (8). The mechanism of NPB-related myopia differs from that of axial myopia in term infants, as NPB-related myopia is produced by abnormal anterior segment components (9, 10). All the above-mentioned events reveal a long-term effect of premature birth on visual impairment, which is also a concern worth addressing.

In prior research, we examined global sex disparity in other non-communicable diseases by analyzing disability-adjusted, life-year data based on the year, age, and socioeconomic status (11–13). Even though NPB-related visual impairment is treatable, the sex disparity in NPB-related visual impairment burden remains a major concern for policymakers who strive to prevent and control blinding eye disease.

In this study, we aimed to explore sex differences in the global burden of vision impairment caused by NPB over time across age groups and countries with different socioeconomic statuses using YLD data from the most recent GBD 2019 Study. The purpose of this paper is to recommend areas for future research to advance our understanding of the interrelationship between sex and vision impairment caused by NPB. In addition, understanding the patterns in vision impairment burden caused by NPB is of great importance for clinicians, researchers, as well as policymakers.

## Methods

### Sex-specific estimates of the burden of vision impairment due to neonatal preterm birth

From 1990 to 2019, data from the GBD 2019 were analyzed to determine the burden of vision impairment caused by NPB (14–16). We searched and extracted associated data, including YLDs numbers, crude rates (adjusted for population size), and age-standardized rates (adjusted for population size and composition), from the GBD group's Internet database (http://ghdx.healthdata.org/gbd-results-tool). The protocol for estimating YLDs was previously published in the GBD 2017 study (17).

The following GBD 2019 data on vision impairment due to NPB were obtained from the Global Health Data Exchange (18): (1) global sex-specific YLD numbers, crude YLD rates (per 100,000 population), and age-standardized YLD rates (per 100,000 population) from 1990 to 2019; (2) global sex- and age-specific YLD numbers and crude rates in 2019; (3) national sex-specific age-standardized YLD rates in 195 countries in 2019; and (4) World Health Organization (WHO) regions' sex-specific age-standardized YLD rates from 1990 to 2019. This study did not require ethics approval or informed consent because the GBD study data are publicly accessible (19).

YLD data allow direct comparisons of NPB-related vision impairment burden by sex from multiple perspectives. YLD is estimated through the number of disability cases multiplied by the disease's average duration and a weight factor that reflects the severity of the disease on a scale from 0 (perfect health) to 1 (dead). Then, YLDs are calculated by multiplying the prevalence of each severity category by severity-specific disability weights (DWs) (20).

There are three types of NPB-related vision impairment: blindness, severe vision loss (SVL), and moderate vision loss (MVL). Based on the Snellen chart, MVL is defined as visual acuity (VA) <6/18 but ≥6/60, SVL as VA <6/60 but ≥3/60, and blindness as VA <3/60 or visual field around central fixation <10% (21).

### National socioeconomic status

As a national socioeconomic indicator, the Human Development Index (HDI) is a measure of average accomplishment in essential aspects of human development, such as living a long and healthy life, being knowledgeable, and having a decent standard of living. The HDI measures socioeconomic development on a scale of 0 to 1, with a greater score indicating a higher level of development. The Human Development Indices and Indicators: 2020 Statistical Update provided HDI data for 2019. Four HDI-based country classifications were introduced in 2019: very high HDI countries (HDI ≥0.804), high HDI countries (0.804 > HDI ≥ 0.703), medium HDI countries (0.703 > HDI ≥ 0.554), and low HDI countries (HDI <0.554).

### Statistical analysis

Wilcoxon signed ranks test was used to analyze the global difference by sex in age-standardized YLD rates and the difference by sex for each HDI-based country group. The association between sex in age-standardized YLD rates and the male to female age-standardized YLD rate ratio with the HDI index was investigated using linear regression analyses. SPSS 26.0 was used for all statistical analyses (IBM, Chicago, USA). Statistical significance was defined as a *p*-value of <0.05.

## Results

### Global burden of NPB-related vision impairment by sex

**Figure 1:**
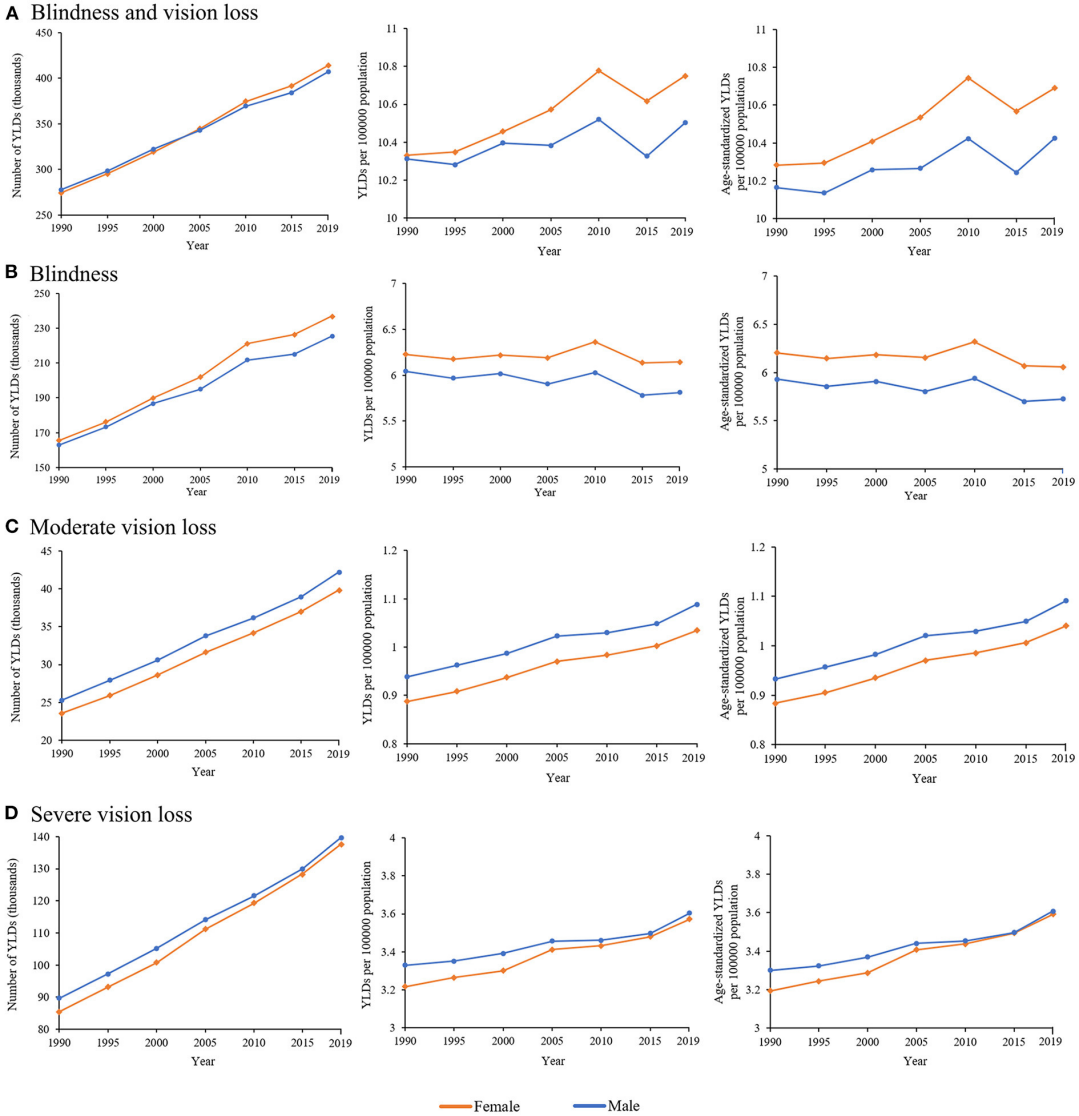
Global sex-specific burden of vision impairment associated with NPB from 1990 to 2019. **(A)** Global burden of NPB-related blindness and vision loss, **(B)** NPB-related blindness, **(C)** NPB-related moderate vision loss, and **(D)** NPB-related severe vision loss in terms of YLD numbers, crude YLD rates, and age-standardized YLD rates. NPB, Neonatal preterm birth; YLDs, years lived with disability; blue line, male; orange line, female.

**Figure 2:**
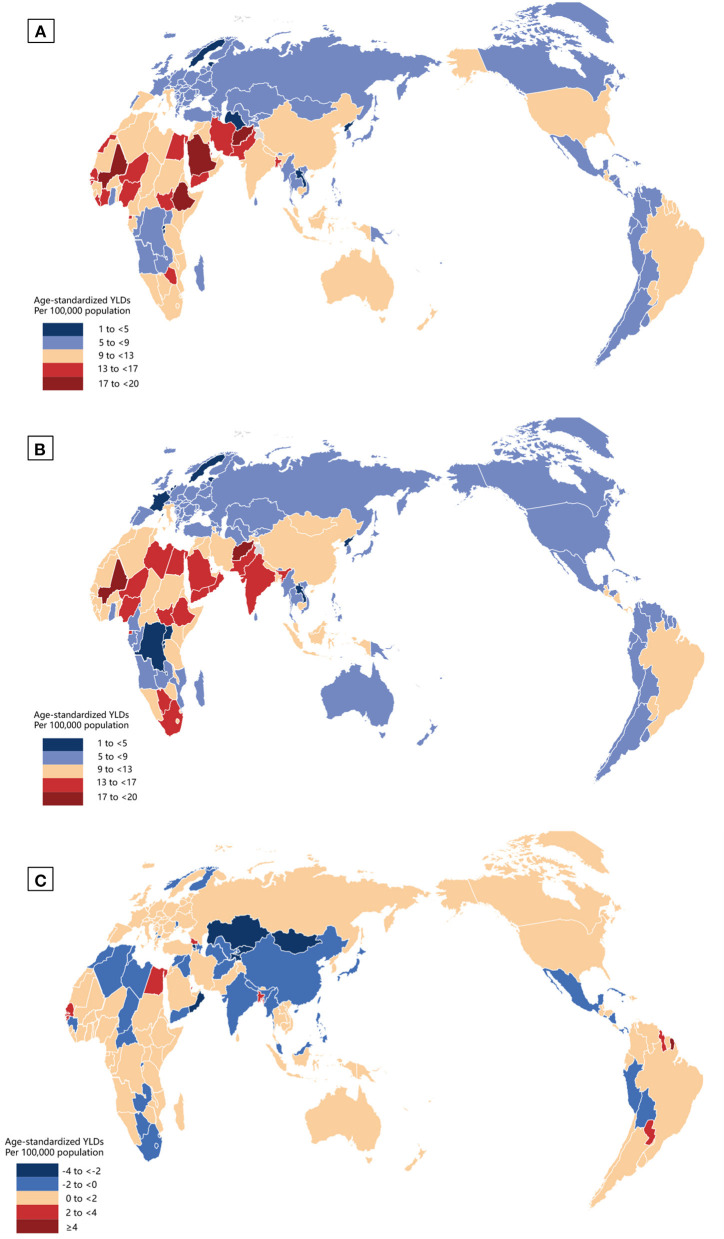
Global map of NPB-related vision impairment burden by sex in 2019. Geographic distribution of age-standardized YLDs per 100,000 population in nations with NPB-related vision impairment in 2019; female **(A)**, male **(B)**, and female minus male **(C)**. NPB, Neonatal preterm birth; YLDs, years lived with disability.

As illustrated in Figure 1, between 1990 and 2019, the differences in YLD numbers of NPB-related vision impairment by sex were notable. Global YLD numbers caused by NPB-related BVL for men rose by 46.8%, from 277.8 thousand [95% uncertainty interval (UI) 183.9 thousand-397.9 thousand] in 1990 to 407.6 thousand (95% UI 270.5 thousand-584.0 thousand) in 2019. Female YLDs caused by NPB-related BVL increased by 51.1% between 1990 and 2019, from 274.4 thousand (95% UI 181.7 thousand-393.5 thousand) in 1990 to 414.6 thousand (95% UI 277.7 thousand-584.1 thousand) in 2019. From 1990 to 2019, females had larger absolute YLD numbers of global NPB-related blindness than males, but men had higher absolute YLD numbers of global NPB-related MVL and SVL. Similarly, differences in rates of NPB-related vision loss by sex were similar between 1990 and 2019. Crude YLD rates caused by NPB-related BVL for men increased by 1.9%, from 10.3 (95% UI 6.8–14.8) in 1990 to 10.5 (95% UI 7.0–10.5) in 2019. Women's YLD rates grew by 4.1% between 1990 and 2019, from 10.3(95% UI 6.8–14.8) in 1990 to 10.8 (95% UI 7.2–15.1) in 2019. After adjusting for population size and age structure, age-standardized YLD rates of NPB-related BVL increased from 10.2 (95% UI 6.7–14.6) in 1990 to 10.4 (95% UI 6.9–15.0) in 2019 among men and from 10.3 (95% UI 6.8–14.7) in 1990 to 10.7 (95% UI 7.2–15.1) in 2019 among women. Moreover, both crude and age-standardized YLD rates of NPB-related BVL and blindness peaked around 2010. Between 1990 and 2019, the gender disparity in terms of NPB-related BVL and blindness remained stable, with women consistently having higher age-standardized YLD rates than males. The gender gap in age-standardized YLD rates of NPB-related MVL remained substantially stable over time, whereas the gender gap in age-standardized YLD rates of NPB-related SVL gradually declined. Figure 2 depicts the global map of NPB-related vision impairment burden by sex for the 195 nations involved in the GBD Study in 2019. Figure 2C shows the NPB-related vision impairment burden of females minus that of males. A paired Wilcoxon test found that females had a greater age-standardized YLD rate of NPB-related BVL (*p* < 0.001), blindness (*p* < 0.001), and SVL (*p* = 0.01), while males had higher age-standardized YLD rates of NPB-related MVL (*p* < 0.001).

### Global sex-specific NPB-related vision impairment burden by age

**Figure 3:**
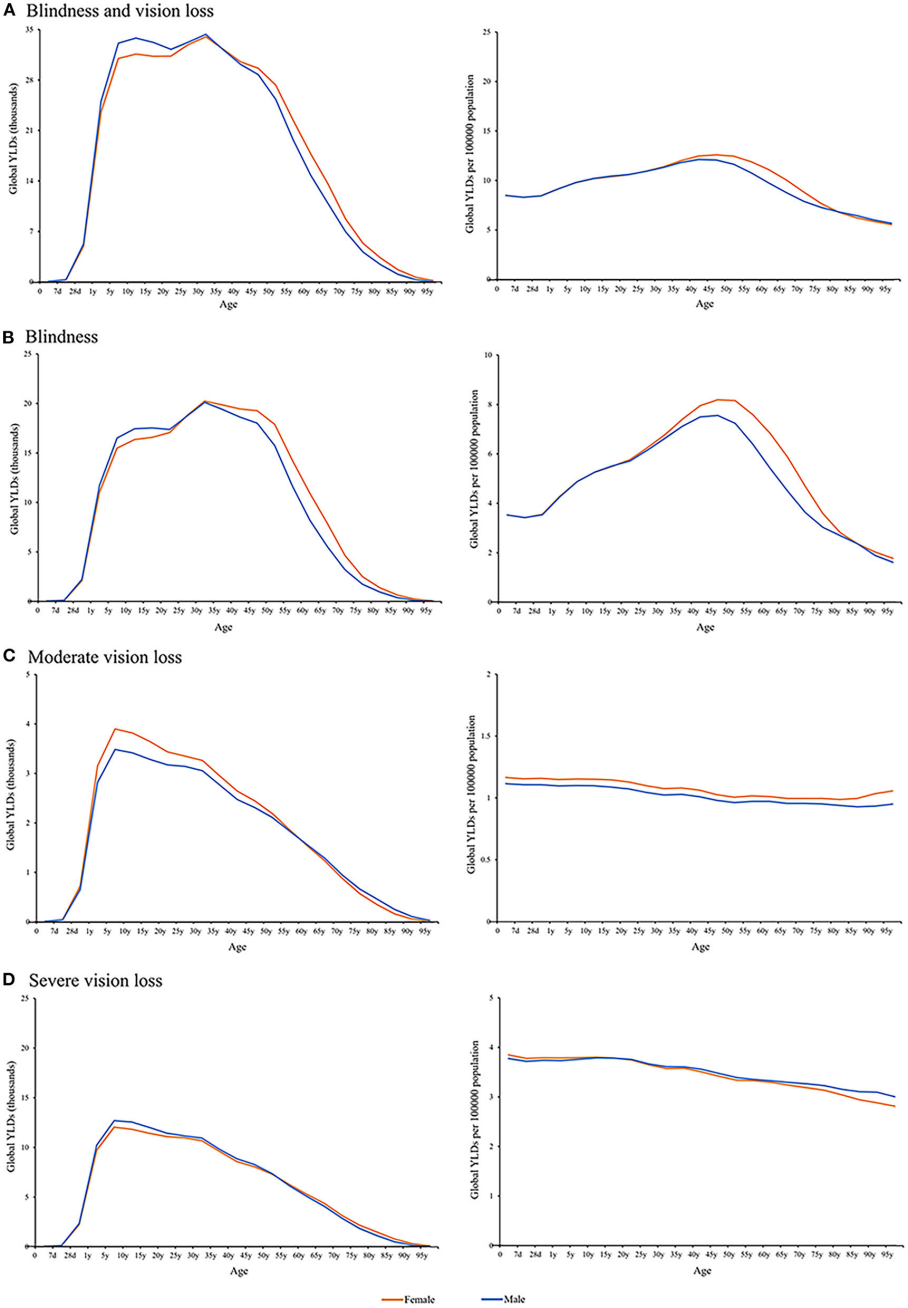
Global sex-specific NPB-related vision impairment burden by age in 2019. Sex disparities in the global burden of **(A)** NPB-related blindness and vision loss, **(B)** NPB-related blindness, **(C)** NPB-related moderate vision loss, and **(D)** NPB-related severe vision loss by age group in 2019, as measured by YLD numbers and crude YLD rates. NPB, Neonatal preterm birth; YLDs, years lived with disability; blue line, male; orange line, female.

GBD study 2019 started to capture vision loss burden due to NPB for persons aged 0–6 days old. Overall, for patients with BVL caused by NPB, YLD numbers increased with age in both sexes from 0 to 39 years old and then decreased in the age group of 95+ years old (Figure 3A). Difference by sex was estimated using absolute difference (female minus male). Males had higher YLD numbers before 35 years. In the 35–39 age group, gender differences were essentially nonexistent. Above 45 years of age, the absolute difference in YLD numbers increased rapidly and peaked in the 60–64 age group (Figure 3A). After adjusting for population size, females exhibited a greater crude YLD rate of NPB-related BVL than males within the range of 25–75 years old (Figure 3A). Sex differences in crude YLD rates (female minus male) were negligibly small until the age of 35 but rapidly increased thereafter. The greatest disparities were reported in NPB-related BVL in the 60–64 age range, with 17,796 YLDs (95% UI 11,872–25,234) in women compared to 14,833 (95% UI 9,995–21,411) in men, and crude YLD rates of 11.10 (95% UI 11.1–15.7) in women compared to 9.75 (95% UI 6.6–14.1) in males.

For NPB-related blindness, females had greater YLD numbers after 25 years and higher crude YLD rates after 20 years. Before the age of 25, there were no significant variations in crude YLD rates between the sexes. The greatest variations in terms of YLDs and YLD rates were reported in NPB-related blindness in the 60–64 age group (Figure 3B). For NPB-related MVL, females had greater crude YLD rates in all age groups and higher YLD numbers before the age of 60 (Figure 3C). For NPB-related SVL, the overall pattern of crude YLD rates was a steady drop with age. Males showed greater crude YLD rates after the age of 20 and higher YLD numbers before the age of 55 (Figure 3D).

### Sex-specific NPB-related vision impairment burden by national socioeconomic status

**Figure 4:**
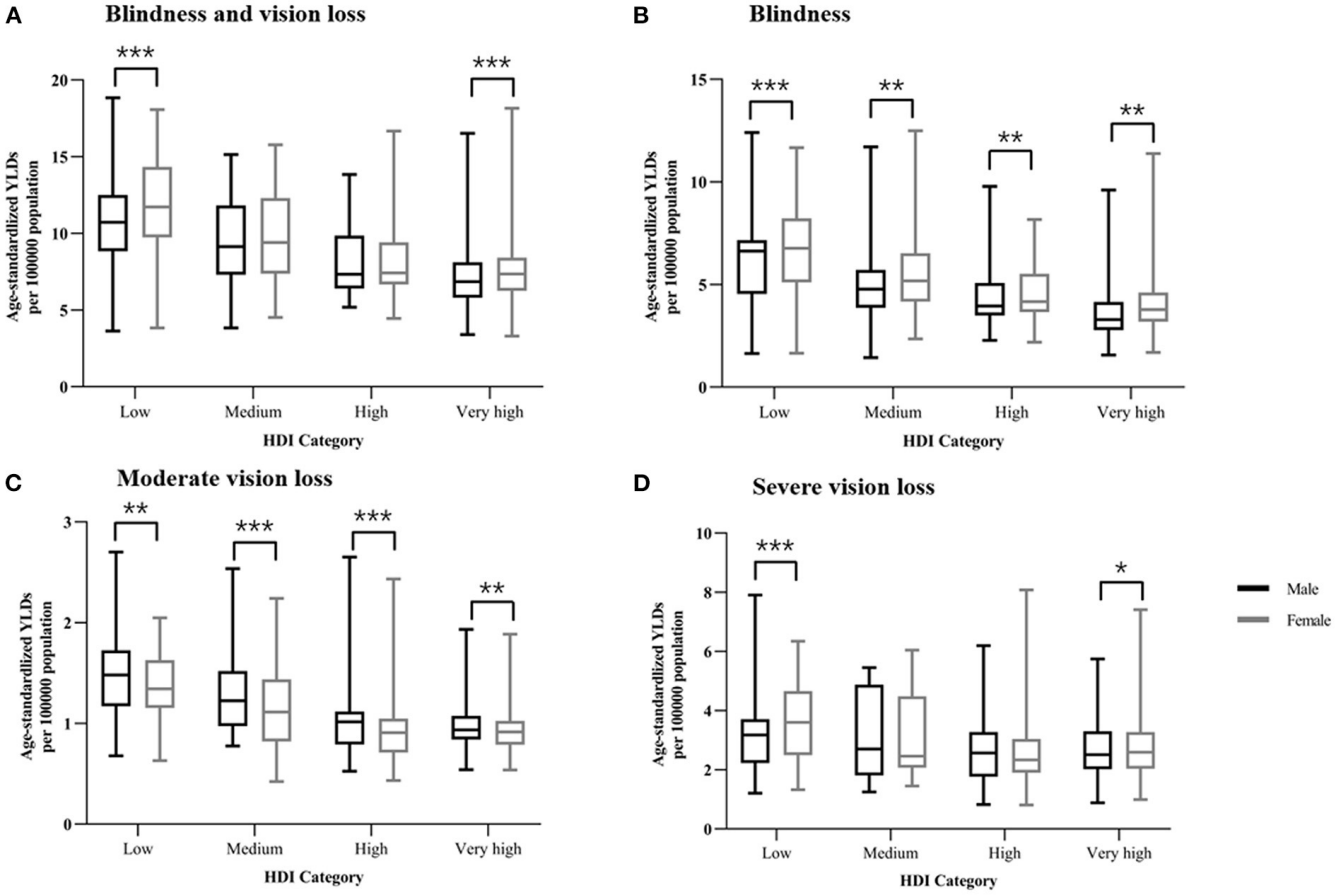
Sex difference of NPB-related vision impairment burden in four socioeconomic development levels. Sex disparities in the burden of **(A)** NPB-related blindness and vision loss, **(B)** NPB-related blindness, **(C)** NPB-related moderate vision loss, and **(D)** NPB-related severe vision loss by each HDI-based country group in 2019, as measured by age-standardized YLD rates. Lines inside the boxes indicate the medians; boxes represent the 25 and 75% percentiles; and lines outside the boxes show the minimum and the maximum values. For difference between sexes, *P < 0.05, **P < 0.01, ***P < 0.001. Wilcoxon signed ranks test. NPB, Neonatal preterm birth; YLDs, years lived with disability; HDI, Human Development Index.

In 2019, HDI data were available for 187 countries, including 64 with a very high HDI, 53 with a high HDI, 37 with a medium HDI, and 33 with a low HDI. In general, lower HDI countries have higher age-standardized YLD rates in both sexes. As illustrated in Figure 4A, age-standardized YLD rates of BVL were higher among females than males in very-high-HDI (*Z* = −4.75, *p* < 0.001) and low-HDI (*Z* = −3.53, *p* < 0.001) countries. For blindness, females had higher age-standardized YLD rates than males in nations with very high HDI (*Z* = −5.55, *p* < 0.001), high HDI (*Z* = −2.46, *p* = 0.014), medium HDI (*Z* = −3.13, *p* = 0.002) and low HDI (*Z* = −3.26, *p* = 0.001) (Figure 4B). In contrast, males had greater age-standardized YLD rates of MVL than females in very-high-HDI (*Z* = −3.41, *p* = 0.001), high-HDI (*Z* = −5.73, *p* < 0.001), medium-HDI (*Z* = −3.55, *p* < 0.001) and low-HDI (*Z* = −2.83, *p* = 0.005) countries (Figure 4C). While for SVL, females had higher age-standardized YLD rates than males in very-high-HDI (*Z* = −2.35, *p* = 0.019) and low-HDI (*Z* = −3.58, *p* < 0.001) countries (Figure 4D).

**Figure 5:**
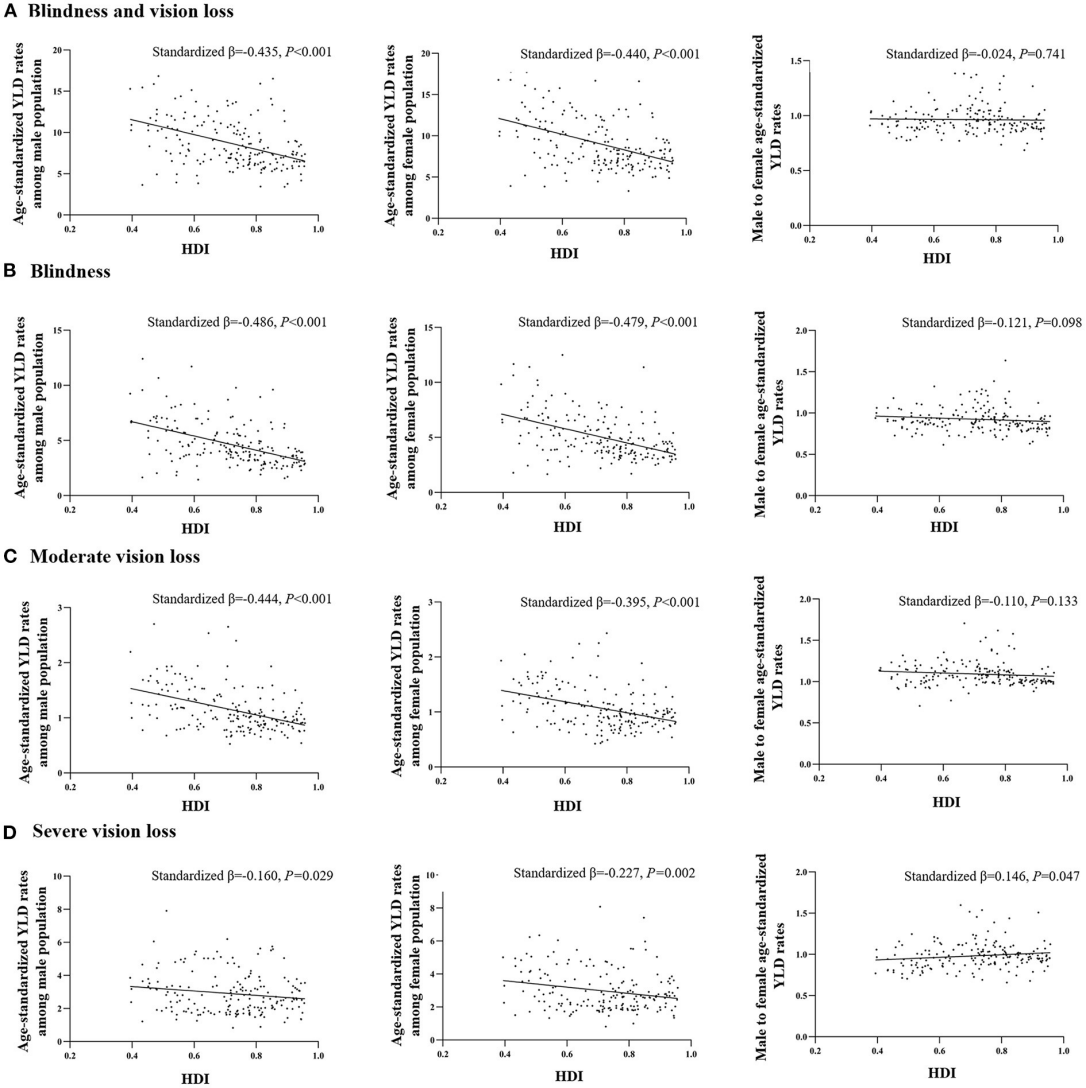
Association between gender difference of age-standardized YLD rates with HDI. The linear relationships between national age-standardized YLD rates in females and HDI, national age-standardized YLD rates in males and HDI, and national female-to-male age-standardized YLD rate ratios and HDI are presented in the following groups: **(A)** NPB-related blindness and vision loss, **(B)** NPB-related blindness, **(C)** NPB-related moderate vision loss, and **(D)** NPB-related severe vision loss. NPB, Neonatal preterm birth; YLDs, years lived with disability; HDI, Human Development Index.

National age-standardized YLD rates in female population (BVL: Standardized β = −0.440, *p* < 0.001; Blindness: Standardized β = −0.479, *p* < 0.001; MVL: Standardized β = −0.395, *p* < 0.001; SVL: Standardized β = −0.227, *p* = 0.002) as well as age-standardized YLD rates in male population (BVL: Standardized β = −0.435, *p* < 0.001; Blindness: Standardized β = −0.486, *p* < 0.001; MVL: Standardized β = −0.444, *p* < 0.001; SVL: Standardized β = −0.160, *p* = 0.029) were negatively related to HDI in linear regression analyses (Figure 5). Male to female age-standardized YLD rate ratios, on the other hand, were positively correlated with HDI in linear regression analyses only for NPB-related SVL (Standardized β = 0.146, *p* = 0.047) (Figure 5).

## Discussion

This study comprehensively revealed the trend of the global burden of vision impairment caused by NPB from 1990 to 2019 with measurements of YLDs and showed that sex differences have persisted in NPB-related vision impairment burden since 1990. Though the absolute total number of NPB-related vision impairment (including blindness, SVL, and MVL) in both sexes displayed increasing trends, the adjusted measurements (including crude and age-standardized rates) peaked around 2010. However, a gender gap in blindness and vision loss has persisted since 1990 and changed little between 1990 and 2019, with women consistently having higher age-standardized YLD rates than men.

Globally, the YLD numbers of blindness, SVL, and MVL showed a rising trend for both men and women. As reported in Zhang et al., the prevalence of blindness, SVL, and MVL due to neonatal disorders increased by 33.53 and 13.73%, respectively, from 1990 to 2019 (22). The NPB-related eye diseases include congenital cataracts, ROP, congenital glaucoma, refractive error, etc. ROP was identified as the primary cause of neonatal disorders related to vision loss, and the prevalence of blindness due to ROP reached 32% by 2019 (22). In this study, while the adjusted measurements of SVL and MVL showed an upward trend, blindness displayed a downward trend with a peak around 2010. The proportion of blindness in all visual impairments YLDs (including blindness, SVL, and MVL) decreased from 59% in 1990 to 56% in 2019. Although blindness still accounted for more than half of the total YLD numbers of visual impairment, there was a declining trend in the YLD rates. This changing trend can be explained by the following reasons. In the past 20 years, the premature infant mortality rates in developing countries had decreased, resulting in an increasing number of survivors (23). Because of the rapid advance in neonatal care, retinopathy of prematurity (ROP) is now the most common cause of vision loss in children and serious cases have declined (4). Since screening premature infants for ROP was advised in 2006, the ophthalmic examination of newborns has increasingly gained prominence and popularity. Thus, the NPB-related blindness and eye diseases, such as cataracts, ROP, and tumors, can be treated in time to avoid irreversible visual impairment. In addition, the application of anti-vascular endothelial growth factor (VEGF) has contributed to a reduction in the ROP-related vision loss. All these measures have led to a reduction in the global NPB-related blindness burden.

Our research observed a gender disparity in NPB-related vision impairment burden by year, age, and socioeconomic regions. Since 1990, there has been a gender gap in blindness and vision loss worldwide, with women continually having higher age-standardized YLD rates than men. Women aged 20–80 have a higher NPB-related blindness burden. Several previous studies have discussed the relationship between sex differences and the incidence of eye disease. Females are significantly more impacted than males by blindness and visual impairment, according to previous research (12, 24). In 2020, an estimated 43.3 million individuals were blind, with 23.9 million of them being female (25). This sex difference may be attributed to a variety of underlying biological, social, cultural, and economic explanations. Previous studies found that severe ROP occurred more often in female infants after 26 weeks of gestation, and this remains after adjusting for perinatal risk factors including IUGR (intrauterine growth restriction), maternal hypertension, use of antenatal steroid, and so on (4). Several studies have suggested that women may suffer from more serious eye diseases, which may explain the gender differences in blindness burden. A report on gender inequities in health problems indicated that most of the eye conditions (including trachoma, cataract, Age-related macular degeneration, glaucoma, vitamin A deficiency, and onchocerciasis) had a female to male excess of 1.2 or more (2). Women are at a higher risk of developing vision impairment or serious eye disease (such as age-related macular degeneration, thyroid eye disease, or chronic dry eye disease) than men (1).

The pattern of blindness and MVL/SVL is not quite the same. The blindness sex difference showed the biggest disparity in the 60 s and the MVL/SVL chart showed the constant difference. There are several reasons for this phenomenon. Previous studies reported that among the global 33.6 million adults aged 50 years and older who were blind in 2020 the leading causes of blindness were cataracts (15.2 million cases), followed by glaucoma (3.6 million cases), undercorrected refractive error (2.3 million cases), age-related macular degeneration (1.8 million cases), and diabetic retinopathy (0.9 million cases) (9). As mentioned in question #3, The onset age of cataracts, age-related macular degeneration, and other diseases is about 60 years old, and blinding eye diseases occur frequently at this age (10, 11). Meanwhile, several studies have reported that sex difference was found and females were preponderance for vision-impairing in cataract, age-related macular degeneration, diabetic retinopathy, glaucoma, and so on (6, 8). Visual function was reduced in prematurely born individuals even in adulthood (12). The reason may be prematurity *per se* since individuals without previous ROP or neurological complications are also affected. Ophthalmological and neurodevelopmental disorders are more common in prematurely born children than in children born at term (13). Previous studies have found a higher prevalence of refractive errors, strabismus, reduced visual acuity, and cerebral visual impairment in preterm adolescents than in full- terms (14, 15). The above reasons may lead to the biggest sex disparity of blindness in the 60 s. We added this part of the discussion and highlighted it.

The sex difference is greater in countries with lower socioeconomic status. Positive relationships between HDI and the female-to-male ratio were observed in SVL by correlating age-standardized YLDs with HDI. The HDI is calculated using the per capita GDP, life expectancy, and literacy rate of a country. The United Nations Development Program (UNDP) proposed the HDI as a broader measure of the development levels in 1990. Although low and medium HDI countries usually bear a heavier burden of reversible eye diseases, our study found a more pronounced sex difference in low HDI and very high HDI countries. This phenomenon may be caused by the following two reasons. First, female newborns are more likely to be neglected in lower socioeconomic level countries. Several studies have reported that lower socioeconomic levels are significantly associated with a higher female disease burden, such as cataracts and uncorrected refractive errors (12, 26). Countries with a lower HDI are reported to have a greater rate of premature births, as well as inadequate nutrition and neonatal care (5, 6). Women's visual medical demands may be underestimated, and women typically lack financial decision-making power, resulting in their inability to pay their visual health bills (27). Besides, lower education levels and literacy prevent women from obtaining and understanding information about vision loss diagnosis and treatments (28). Second, women's relatively longer life expectancy in developed countries can partially scale up the sex difference of NPB-related vision impairment burden. A previous study predicted a sex difference in life expectancy across 54 nations and found that females have a 5.8-year lead over males in terms of life expectancy at birth (29). Women live longer than men, resulting in their more years living with a disability than their male counterparts.

There are still some limitations in our study. Firstly, we calculated all diseases that may lead to visual impairment but not according to the classification of diseases. Therefore, we could only obtain an overall impression instead of refining the data into various diseases. Secondly, our study was affected by flaws in the 2019 GBD study methodology, and the bias in the original data sources can affect our statistical hypotheses. Bias might come from the use of aggregate data at a national level instead of district data, because geographic variations in YLDs estimates may occur. Although this study provided a global view of differences in NPB-related visual impairment burden by sex, the conclusions may not be applicable to a specific district. Although our study revealed sex differences and global socioeconomic in the burden of vision impairment associated with neonatal preterm birth, this conclusion needs to be interpreted with caution in specific regions. As the GBD study updates, we also need to keep an eye on the long-term impact of a visual burden.

## Conclusion

Although global NPB health care is progressing, sexual differences in NPB-related vision impairment burden have shown little improvement in the past few decades. Worldwide, females bear a greater NPB-related vision impairment burden than males. Greater sex differences were found in low and very high socioeconomic status countries. Our findings may enhance public awareness of sexual differences in global NPB-related vision impairment burden and emphasize the importance of making sex-sensitive health policies to manage global vision loss caused by NPB.

## Data availability statement

The raw data supporting the conclusions of this article will be made available by the authors, without undue reservation.

## Ethics statement

Ethical review and approval was not required for the study on human participants in accordance with the local legislation and institutional requirements. Written informed consent from the patients/participants or patients/participants' legal guardian/next of kin was not required to participate in this study in accordance with the national legislation and the institutional requirements.

## Author contributions

XY had full access to all the data in the study, will take responsibility for the integrity of the data, the accuracy of the data analysis, and study concept and design. XY and JW: acquisition, analysis, or interpretation of data. XY, JW, XZ, WQ, SY, and SH: drafting of the manuscript. XY and LL: critical revision of the manuscript for important intellectual content. LL and LS: study supervision. All authors contributed to the article and approved the submitted version.

## Conflict of interest

The authors declare that the research was conducted in the absence of any commercial or financial relationships that could be construed as a potential conflict of interest.

## Publisher's note

All claims expressed in this article are solely those of the authors and do not necessarily represent those of their affiliated organizations, or those of the publisher, the editors and the reviewers. Any product that may be evaluated in this article, or claim that may be made by its manufacturer, is not guaranteed or endorsed by the publisher.
